# Current and Future Repellent Technologies: The Potential of Spatial Repellents and Their Place in Mosquito-Borne Disease Control

**DOI:** 10.3390/ijerph14020124

**Published:** 2017-01-29

**Authors:** Edmund J. Norris, Joel R. Coats

**Affiliations:** Department of Entomology, Iowa State University, Ames, IA 50011, USA; ejnorris@iastate.edu

**Keywords:** repellent, DEET, para-menthane-3,8-diol, mosquito, disease, pyrethroid, biorational, spatial, contact, resistance

## Abstract

Every year, approximately 700,000 people die from complications associated with etiologic disease agents transmitted by mosquitoes. While insecticide-based vector control strategies are important for the management of mosquito-borne diseases, insecticide-resistance and other logistical hurdles may lower the efficacy of this approach, especially in developing countries. Repellent technologies represent another fundamental aspect of preventing mosquito-borne disease transmission. Among these technologies, spatial repellents are promising alternatives to the currently utilized contact repellents and may significantly aid in the prevention of mosquito-borne disease if properly incorporated into integrated pest management approaches. As their deployment would not rely on prohibitively expensive or impractical novel accessory technologies and resources, they have potential utility in developing countries where the burden of mosquito-borne disease is most prevalent. This review aims to describe the history of various repellent technologies, highlight the potential of repellent technologies in preventing the spread of mosquito-borne disease, and discuss currently known mechanisms that confer resistance to current contact and spatial repellents, which may lead to the failures of these repellents. In the subsequent section, current and future research projects aimed at exploring long-lasting non-pyrethroid spatial repellent molecules along with new paradigms and rationale for their development will be discussed.

## 1. Introduction

Mosquitoes represent one of the most significant threats to human and veterinary health throughout the world. With over 3500 unique species of mosquito currently described that inhabit diverse ecosystems and feed on a variety of host species, their ubiquity and capacity to transmit disease agents are unparalleled in the animal kingdom [1]. Moreover, their coevolution with disease agents, reservoir hosts, and human communities has endowed them with an exquisite ability to vector particularly successful and debilitating human and veterinary pathogens. The parasites and viruses that are vectored by mosquitoes are responsible for the death of several hundred thousand people each year, with hundreds of millions more infected with debilitating consequences. Moreover, this disease burden is most substantial in developing countries, which lack the infrastructure and economic and educational resources necessary to properly control the spread of mosquito-borne disease [2]. 

In general, there are two primary methods used to control the spread of mosquito-borne disease. Targeting the disease agent is an often-utilized and readily exploitable means of preventing its transmission to a subsequent human host. These strategies include prophylactic measures, mass drug administration campaigns, vaccinations, and antibiotic drugs and antivirals that selectively target the parasite or inhibit the replication of the arbovirus in the host [3]. While extremely successful in the control of certain etiologic disease agents, such as filarial parasites and some strains of *Plasmodium*, resistance of parasites to various drugs and the lack of antivirals and vaccines for many disease agents limit the success of this control technique for many mosquito-borne disease agents [4,5,6,7]. The second method of control involves the prevention of transmission of the disease agent by the vector. This is accomplished by a variety of means including the abatement of vector populations and the use of biting deterrents. While vector control has been an extremely successful method in limiting the spread of mosquito-borne disease throughout the world, the advent of insecticide-resistance in many wild mosquito populations threatens the effectiveness of this approach in the future [8]. As more of the limited insecticidal classes approved for use against public health pests fail in controlling mosquito populations, the need for new chemistries and control strategies becomes ever more paramount.

A number of new mosquito control strategies have become more widely discussed, tested and exploited in recent years; however, the likelihood of these strategies succeeding in field scenarios has yet to be determined. These strategies primarily focus on limiting the numbers of actively host-seeking female mosquitoes in the wild. Among these novel control strategies are the use of attractive toxic sugar baits, mass-trapping techniques, auto-dissemination of hormone mimetics, push-pull strategies, and the release of *Wolbachia*-infected or irradiated sterile males/genetically modified individuals into the field [9,10,11,12,13,14,15]. While these strategies certainly represent important novel methods with potential for limiting the spread of vector-borne disease, numerous hurdles still prevent their deployment in large enough regimens to allow for adequate control of wild mosquito populations. Toxicity to non-target organisms, logistical deployment obstacles, the cost of these novel strategies, and public distrust of mass release protocols involving genetically modified mosquitoes are all poignant issues that will need to be addressed before these methods may be readily and efficiently utilized.

Novel repellent tactics represent promising alternatives in preventing the spread of mosquito-borne disease without the logistical hurdles of the aforementioned novel mosquito control approaches. Among these, spatial repellents represent an exciting means by which host-seeking female mosquitoes may be deterred from entering residences and feeding on susceptible individuals. Moreover, their deployment would not rely on the use of currently non-existent or widely unavailable technologies as some of the more ambitious novel control methods (e.g., mass-rearing facilities for the production of genetically modified insects, deployment of large numbers of traps, etc.). Instead, spatial repellents can readily be deployed with current technologies and with relatively little cost [16]. As such, this strategy may be ideal in developing nations where vector control resources or funds are limited. 

In the following sections, we will briefly describe the history of repellent technologies, the role of repellent technologies in the prevention of mosquito-borne disease transmission, the unique characteristics of spatial repellents compared to contact repellents, resistance to current contact and spatial repellent technologies, and currently utilized spatial repellent technologies and the future of this approach. This review will provide background on this strategy and discuss the prospect of this approach in future integrated pest management practices aimed at reducing the spread of debilitating mosquito-borne illnesses. Finally, research projects aimed at the development of novel spatial repellent chemistries and the rationale employed and the physicochemical paradigms exploited in their development will be discussed.

## 2. A Brief History of Repellents

The act of repelling biting arthropods is not a modern approach. Documented attempts of deterring hematophagous insects date back to antiquity. Among the earliest reports of repellent use are from Herodotus, a Greek historian [17]. His account of communities burning plants to prevent the aggregation of biting flies demonstrates the success of this strategy. Before synthetic chemistry approaches, botanical extracts and mechanical barriers constituted the primary means by which individuals prevented bites from arthropods. Among the most successful plant extracts initially used for the prevention of mosquito bites were citronella, cassia, cedar, lavender, eucalyptus and neem tree oil [18,19].

The advent of World War II was the primary driver in the development of new repellent technologies, as the Pacific and North African theaters posed significant disease threats to Allied military personnel. The testing of over 6000 chemicals from 1942 to 1947 in a variety of research institutions led to the identification of multiple successful repellent chemistries [20,21,22,23]. This work established a number of independent research projects that inevitably identified one of the most effective and widely used insect repellents to date, *N*,*N*-diethyl-*meta*-toluamide (DEET). Since then, a number of other compounds have been synthesized that heavily relied on previous research, which identified amide and imide compounds as highly successful contact repellents. Among these are picaridin, a piperidine carboxylate ester, and IR3535, which are currently considered to rival DEET in some repellency bioassays [24]. 

While synthetic repellent chemistries have shown to be highly effective since the mid-20th century, newly discovered natural chemistries are also highly effective. Para-menthane-3,8-diol represents a naturally derived repellent compound from the oil of lemon eucalyptus that has been officially recognized by the Centers for Disease Control (CDC) [25]. The paucity of CDC recommended and Environmental Protection Agency (EPA) approved plant-based repellents is largely based on their relatively short-lived residual character on treated surfaces when compared to the less volatile synthetic repellents, DEET and picaridin [26]. 

However, many researchers have demonstrated the efficacy of botanical compounds as insect repellents [27,28,29]. These reports have characterized numerous plant compounds as potent repellents to a wide variety of pestiferous insects. These chemistries represent potential in the development of novel repellent approaches, via improved formulations or novel synthetic approaches based off of original botanically derived chemistries. These biorational synthetic approaches may increase the repellent and residual character of these compounds on treated surfaces and represent exciting areas of active research. By identifying the physicochemical properties that contribute to high levels of repellency and long-lasting residual character on treated surfaces, new, highly efficacious spatial and contact repellent technologies may be brought to market. 

## 3. The Role of Repellents in Disease Prevention

**Figure 1:**
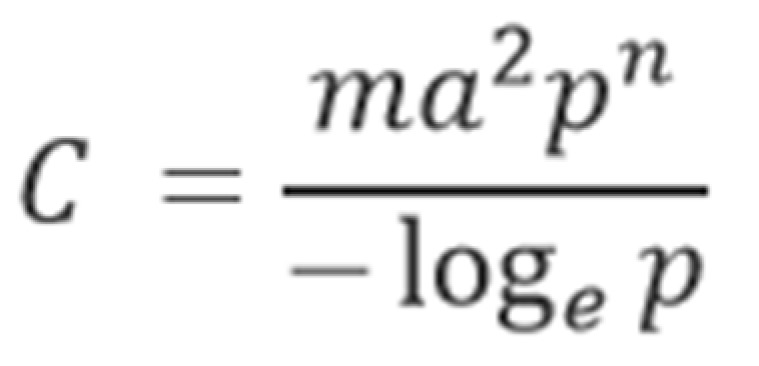
Equation describing the vectorial capacity of a particular vector population. *m* = mosquito abundance, *a* = interaction describing the man-biting rate, *p* = probability of daily mosquito survival, *n* = extrinsic incubation period (EIP) in days [30].

The interruption of host-seeking is the cornerstone of all arthropod disease vector control programs. Even approaches aimed at curtailing wild vector populations inevitably rely on the prevention of host-seeking or biting as an endpoint. Vectorial capacity is a measure of the effectiveness of a given vector species to transmit a particular etiologic disease agent. This metric is reliant on a number of factors that either increase or decrease the likelihood of an arthropod vector to successfully disseminate biologically viable disease agents to their target host species [30]. Because mosquito-biting rates represent a second-order parameter in overall vector capacity, it is theoretically possible to drastically lower the spread of mosquito-borne disease by disrupting host-seeking and feeding (Figure 1). Therefore, repellents represent an important tool in the fight against mosquito-borne disease. Unfortunately, their place in official integrated pest management approaches have been largely neglected.

Numerous studies have attempted to characterize the effects of repellent chemistries on the mitigation of arthropod disease transmission [31,32,33]. The majority of these studies utilize modeling approaches, which aim to characterize the effects of repellent usage in an isolated, albeit artificial, scenario. These studies have repeatedly reported the promising potential of repellents in preventing the spread of mosquito-borne disease. Various models have attempted to characterize the impact of repellents on the presence of mosquitoes in treated areas and relate this to disease transmission. Lutambi et al. demonstrated that spatial repellents were capable of preventing mosquito entry into particular areas if mosquito sources were below a certain threshold. Lower travel distances for mosquitoes in treated compared to untreated control regions may also limit the ability of mosquitoes to complete aspects of their life cycle (e.g., oviposition, mating, blood-feeding) and thus contribute to an even higher negative impact on overall disease transmission [34]. 

While topical repellent technologies can be somewhat successful in limiting cases of mosquito-borne disease in endemic regions, their efficacy in preventing mosquito-borne disease is still currently under debate from an epidemiological perspective. In field studies, the effect of repellents on the biting rates of mosquitoes is significant. In a double-blind randomized cross-over placebo-controlled study in Senegal, repellents composed of DEET, *para*-menthane-3,8-diol, and picaridin all provided significant biting protection on exposed human subjects compared to the control group when biting rates were monitored for 9 h after exposure to topical repellents [35]. However, it is not known whether this temporary decrease in biting rate could translate to decreased transmission of mosquito-borne disease. A six-month field trial in Pakistan indicated repellent soap containing DEET was highly successful in limiting *Plasmodium falciparum* cases compared to the control [36]. Cluster-randomized, placebo-controlled trials in Tanzania reported a non-significant decrease in the malaria cases observed in the contact repellent group compared to the placebo [37]. However, authors reported that both low compliance and differences in socioeconomic statuses of the treatment and control group may have confounded the results in this study. These conflicting results demonstrate that much is still to be learned about the value of contact repellents from an integrated pest management strategy aimed at preventing the spread of mosquito-borne disease.

A commonly reported logistic hurdle with the deployment of contact repellents is the failure of susceptible individuals to reapply at recommended time intervals [37,38,39]. For example, even the most highly concentrated commercial formulations of DEET need to be reapplied every several hours in order to adequately prevent mosquito bites. This can be impractical or impossible in regions where supplies are limited or education paradigms face challenges in instilling the importance of this recommendation to community members. Moreover, in many tropical areas where mosquito-borne diseases are endemic, high levels of perspiration can further limit the recommended reapplication interval, making this approach more impractical [40]. As such, chemical repellent strategies need to be re-envisioned, to integrate the lifestyles and resources available to many susceptible individuals in mosquito-borne disease endemic areas. Spatial repellents offer great potential in this respect. Wada et al. demonstrated that metofluthrin-impregnated lattices were capable of preventing the resting of *Ae. aegypti* within residences for a minimum of 8 weeks after introduction. This study demonstrates the potential of this approach in curtailing mosquito entry into living quarters [41] for relatively long periods of time compared to the protection afforded by current contact repellent technologies. 

The benefit of these technologies needs to be adequately quantified via large scale epidemiological comparisons of regions utilizing these chemistries compared to those which are not. To date, no studies of this kind have been completed. However, numerous research projects are underway which aim to characterize their effects in real-world, public-health-focused trials. Kawada et al. demonstrated that metofluthrin-impregnated plastic strips caused statistically significant repellency of *Anopheles gambiae* (*sensu lato*) from entering rural houses in coastal Tanzania for >18 weeks after introduction [41]. While this study does not relate the decrease of mosquito density in homes to the occurrence of mosquito-borne disease, it represents the promise of spatial repellents as a novel intervention strategy. Recently, SC Johnson announced the expansion of WOW™, a business model that attempts to provide pest control and repellent technologies alongside home cleaning products in an attempt to combat disease transmission in malaria-endemic regions [42]. This model utilizes transfluthrin applied to a surface, such as a wall-mounted poster or substrate, in a passive-emanation approach. The long-term effects of this campaign on both mosquito density in homes and the subsequent impact on disease transmission will need to be evaluated in the years to come. 

## 4. Spatial vs. Contact Repellents: Similarities and Differences

Repellent technologies are similar in regards to their ability to prevent mosquitoes from feeding on susceptible individuals. However, the modes by which these chemistries exert their effects are unique. Biting deterrence may be accomplished by a variety of means including the disruption or interference of proper host-seeking, altering the locomotion of the insect, or causing mortality when in contact with repellent compounds. All of these responses disrupt the ability of the vector to successfully contact and feed on the host, resulting in successful repellency. The responses of mosquitoes to these various compounds is a function of both the physiological responses elicited by these compounds and the physicochemical properties of these repellent molecules. 

The physiological responses of the most commonly utilized contact repellent, DEET, have been investigated in numerous studies, and the elucidation of its mechanism of action has been complex. Results from various behavioral assays indicate that DEET is capable of decreasing attraction caused by various host odorants, such as *L*-lactic acid and 1-octen-3-ol [43,44,45,46]. These studies, in concert, led to the hypothesis that DEET was capable of attenuating the antennal responses of mosquitoes and other hemotophagous insects to various human and veterinary attractive odorants via direct inhibition or attenuation of action potential amplitudes or frequencies emanating from olfactory receptor neurons (ORN). While this hypothesis has been substantiated by changes in direct antennal recordings after exposure to DEET [43], a number of other studies have elucidated a more complex physiological mechanism for the perception of DEET by host-seeking female mosquitoes. DEET was shown to decrease the attractancy of mosquitoes to a simple stimulus of carbon dioxide. This observation suggests that DEET was not only capable of attenuating the perception of host organic volatiles, but also of inorganic carbon dioxide, as well. The current understanding of how DEET causes significant decreases in mosquito host-seeking is that its perception by antennal and palpal sensilla is mapped to an aversive behavioral response. Syed et al. demonstrated that mosquito antennal sensilla were capable of detecting DEET alone. This combined with the observed aversion of both male and female mosquitoes to a sucrose/water-only treatment indicated that the mechanism of DEET did not include the masking of various organic and inorganic host-seeking volatiles, but instead, repellency was due to direct stimulation of antennal sensillae and subsequent activation of an aversive response. These results are further corroborated by the identification of an odorant receptor, DmOr42a, from *Drosophila melanogaster* which was capable of binding to DEET, picaridin, and IR3535, leading to a subsequent action potential. This study also demonstrated the behavioral aversion of fruit flies to exposures of DEET alone [47]. Other studies have also shown that DEET is capable of directly attenuating the antennal responses of mosquito antennae to host volatiles. Ditzen et al. showed that DEET was capable of directly decreasing the antennal response of *An. gambiae* to both CO_2_ and 1-octen-3-ol when exposed to both DEET and odorant compounds simultaneously [48]. These studies in concert suggest that DEET is capable of causing both an aversive stimulus and masking the perception of host odors. Many other studies have demonstrated that the piperidinecarboxylate picaridin and aminopropanoate IR3535 function in a similar manner, but with varying efficacies [49]. As such, these compounds have served as successful insect repellents for years. Their physicochemical properties, however, directly affect their utility and limit their use in certain exposure methods. 

DEET, picaridin, and IR3535 are all classified as contact repellents [50,51]. Because of their low volatility, these compounds remain on treated surfaces and do not volatilize to an appreciable degree into the surrounding atmosphere. Multiple studies have demonstrated their longevity on treated surfaces [52,53]. In order for these compounds to be effective, mosquitoes must come into close proximity or direct contact with the treated surface in order to be repelled from an attractive source. While this is a successful means of exposing mosquitoes to repellent compounds, it requires that susceptible individuals reapply these contact repellents to their skin or the skin of susceptible animal hosts after their volatilization or absorption into the skin, which limits their effectiveness. This may be impractical in certain environments where reapplication is difficult or must occur more frequently. Spatial repellents offer an alternative to this approach and allow for the continual emanation of repellent compounds into the air. 

In general, spatial repellent compounds are highly volatile and capable of diffusing through the air in treated regions [54]. The volatilization of these compounds creates repellent vapors that host-seeking mosquitoes may come into contact with, leading to an aversive behavior or deleterious physiological response from the vector. Currently marketed spatial repellent products utilize either synthetic pyrethroids or botanical compounds. Through the use of passive emanators, candles, burned coils, heating elements, and motorized fans, these compounds may be directly volatilized into the area around susceptible individuals or host animals [16,55]. Studies performed on the mechanism of action of pyrethroid-based spatial repellents are numerous. Because the ultimate goal of repellent technologies is to deter host-blood-feeding, these compounds may exert their effects through a variety of means. Transfluthrin and metofluthrin were capable of deterring overall mosquito entry into treated huts and inhibited feeding, decreased fecundity, and higher percentage mortalities were observed in mosquito groups that entered these treated huts [56]. Other studies have also demonstrated the sublethal effects of spatial repellents on mosquitoes that have been exposed to these repellent vapors. While the physiological responses caused by these compounds have not been fully elucidated, it has been demonstrated that antennal perception of these pyrethroid compounds may be involved [57]. Although the molecular mechanisms behind the mode of action of synthetic pyrethroid spatial repellents still needs to be explored and further characterized, the modes of action of these spatial repellents may be particularly important for an integrated pest management strategy. 

Botanical compounds exert similar effects to synthetic pyrethroid spatial repellents, however, the physiological responses of exposed mosquitoes that mediate these effects are different. Whereas spatial repellent pyrethroids are most likely perceived by a small subset of antennal receptors, the physiological perception of botanical compounds is most certainly mediated via the binding of these compounds to numerous odorant binding receptors in mosquito antennae. Terpenoid compounds were shown to increase antennal spike frequency in *Culex quinquefasciatus* females exposed to various plant terpenoids. The spike frequencies caused by terpenoids were greater than those caused by other repellent compounds, such as diethyl phthalate [58]. Moreover, numerous odorant receptors were identified in *An. gambiae* after transfection into empty neuron systems that bound various terpenoid molecules and elicited increases in antennal spike frequencies [59]. The behavioral responses of mosquitoes exposed to these chemistries is similar to synthetic spatial pyrethroids. Mosquitoes exposed to various plant essential oils experienced significant repellency away from treated regions. Much like spatial repellent pyrethroids, lethal effects of these plant essential oil repellent treatments were also noted [60]. It is possible that botanically derived compounds may exert much the same effects as synthetic pyrethroid spatial repellents. Because of this, they may represent promising alternatives to synthetic pyrethroid spatial repellents without the potential of cross-resistance with these molecules. The lack of structural similarity between most botanically derived compounds and synthetic pyrethroid spatial repellents may indicate that distinct odorant receptors are involved in the perception of these separate chemistries. However, the similarity in the behavioral responses elicited by these two chemical classes provides evidence that both may be useful in future integrated pest control management strategies that incorporate spatial repellents in their paradigm. 

## 5. Resistance to Repellent Chemistries

While repellent pyrethroid and benzamide chemistries represent promising tools in the prevention of mosquito-borne disease to both humans and animals, resistance (or insensitivity) to these chemistries is developing and comprises a significant hurdle in the development and deployment of these strategies in the future. In general, resistance to both contact repellents and synthetic pyrethroid spatial repellents has been reported. While the physiological mechanisms behind these different types of resistance are still being elucidated, preliminary studies have demonstrated that these resistant phenotypes are distinct and unique. 

Behavioral and physiological resistance to contact repellents have been reported in a number of laboratory studies and selection of repellent insensitive colonies has been achieved. Vinauger et al. showed that *Aedes aegypti* females were less averse to DEET-treated substrates if they had been exposed to DEET previously, indicating that a behavioral desensitization to continual exposures to DEET may occur in wild mosquito populations [61]. Pellegrino et al. demonstrated that individual fruit flies that were insensitive to DEET possessed a single amino acid polymorphism compared to the DEET sensitive group [62]. This single amino acid polymorphism, located in the Or59b, leads to lack of feeding inhibition upon exposure to DEET. This prevents DEET from masking host odors used in the perception of susceptible individuals and animals. This study, in concert with another previously mentioned [47], demonstrates that multiple receptors are responsible for the perception of DEET in fruit flies. Moreover, mutations in odorant receptors capable of perceiving DEET may be sufficient to allow for insensitivity to DEET. DeGennaro et al. demonstrated that specific mutations achieved via targeted zinc finger nucleases in *orco*, an obligate co-receptor required for the perception of many host and non-host odors, resulted in a lack of sensitivity to DEET [63]. While this obligate co-receptor is important in normal host-feeding, its role in the development of resistance to DEET has yet to be determined in wild field populations. A loss-of-function mutation in this receptor may lead to significant fitness costs and may not confer resistance to repellents in future field populations of mosquitoes. Another study noted increased insensitivity of antennal sensilla via continual laboratory selection of DEET-insensitive *Ae. aegypti* females after nine generations compared to the unselected control colony [64]. This study further characterized the trait as dominant in its inheritance. These studies indicate that contact repellent resistance to benzamide compounds, such as DEET, are inheritable and selectable. The prevalence of DEET insensitivity in wild mosquito populations has yet to be determined, however, insensitivity to DEET and other contact repellents in wild field populations is possible. Populations of *An. albimanus* from Belize and El Salvador displayed different susceptibilities to DEET and a piperidine analog of picaridin, SS220 [65]. This report demonstrates that intrinsic or selected differences in repellent sensitivities may exist in wild mosquito populations. As mosquito repellents become more utilized in integrated pest management control measures, the issue of resistance to various contact repellents may become more important in the years to come. 

Resistance to synthetic pyrethroid spatial repellents is now being explored in laboratory colonies. Because of the prevalence of pyrethroid resistant wild mosquito populations, the likelihood of resistance to synthetic pyrethroid spatial repellents in wild mosquito populations is quite high. In a recent study, laboratory colonies that were selectively bred for multiple generations based on their non-responsiveness to transfluthrin in a spatial repellency bioassay were more likely to be less susceptible to the repellent effects of transfluthrin in subsequent generations. Later, the toxicity of transluthrin to these selected mosquitoes was evaluated via CDC bottle bioassay, resulting in lower toxicity in the selected group and higher knockdown resistance (*kdr*) allele frequencies [66]. This study indicates the potential of extant pyrethroid resistance in wild mosquito populations conferring resistance to these spatial repellents. Other groups have also demonstrated that the *kdr* allele confers resistance to pyrethroids as repellents. Agramonte et al. demonstrated that a *kdr* Puerto Rican strain of *Ae. aegypti* were less repelled by permethrin impregnated clothing [67]. Moreover, *kdr* mosquitoes collected from field sites in Puerto Rico were shown to be less susceptible to synthetic pyrethroids in a spatial repellent bioassay compared to control (susceptible) mosquitoes [49]. These studies demonstrate the potential for the development of resistance to synthetic pyrethroid spatial repellents. Because of the reliance on pyrethroids in the control of wild mosquito vector populations and the concomitant development of pyrethroid-resistance, it is likely that the deployment of synthetic pyrethroid spatial repellents will be met with efficacy concerns in regions where extant pyrethroid resistance is prevalent. 

Because of resistance to both contact and synthetic pyrethroid spatial repellent compounds, new chemistries need to be utilized in future repellent technologies. Botanical compounds offer great potential for the purposes of developing new repellents against pyrethroid-resistant mosquitoes. A variety of odorant receptors have been demonstrated to bind and respond to multiple botanical compounds [59]. Among 50 odorant binding receptors isolated from *An. gambiae* expressed in empty neuron systems, multiple receptors were responsive to terpenes and terpenoid ketone compounds (carvone and fenchone). The authors also describe that many odorant receptors were not screened in this study, indicating that many more receptors may be involved in the detection of botanical repellent compounds. Moreover, their efficacy as repellents and the safety of these compounds to mammals has been shown in multiple examinations [68]. The large number of odorant receptors implicated in the perception of these botanical compounds in both mosquitoes and fruit flies [69] may indicate that resistance to these compounds may evolve more slowly than to other synthetic pyrethroid spatial repellents. Instead of a specific single receptor implicated in the perception of these compounds in mosquito antennae, the presence of multiple receptors may make it difficult for single mutation events to lead to resistance to these compounds. Moreover, the repellency of botanical compounds may be heavily correlated with their fumigant toxicity [70]. The mechanism of action behind the toxicity of various plant compounds is complex and diverse. The mechanism of toxic action of various terpenoid compounds include inhibition of acetylcholinesterase, activity at octopamine and tyramine receptors, nicotinic acetylcholine receptor activity, and modulation of GABA-gated chloride receptors in insects [2]. Because of these diverse mechanisms of action and the potential of causing repellency via both antennal perception and fumigant toxicity, resistance to botanical compounds utilized as future repellent technologies may occur more slowly than toward synthetic contact and spatial repellents. These chemistries may represent some of the most promising leads for the development of repellents for the prevention of mosquito-borne disease transmission. 

## 6. Current and Future Technologies

There are a variety of contact repellent products currently on the market which have been recommended by the Centers for Disease Control and the Environmental Protection Agency in preventing arthropod-borne disease [71]. These recommendations are primarily made based on the residual character of these compounds on treated surfaces or human skin. These products include DEET, picaridin, and *para*-menthane-3,8-diol. Numerous products containing DEET are used and considered to be highly efficacious as contact repellents and provide repellency to a wide range of hematophagous insects. These products provide repellency for one to eight hours (depending on concentration) after their application. Although these persistence times are considered long-lasting with respect to other products that are currently on the market, there is still significant room for improvement in repellent technologies. 

With respect to spatial repellents, numerous products are available. These include transfluthrin, metafluthrin, and various botanical oils or compounds (e.g., oils of citronella, peppermint, lemongrass) volatilized into a head space with the goal of repelling biting mosquitoes [54]. In recent years, these products have become significantly more common, and the potential of their deployment in large scale disease prevention campaigns throughout the world is becoming more feasible [16]. However, due to the potential of resistance to various old and currently utilized repellent technologies, new chemistries and strategies must be developed and employed. The modification of botanical compounds also represents a very promising avenue in the development of new, long-lasting, and safe repellent technologies. 

Botanical repellent technologies have been a promising area of research for many years. Their efficacy and safety to mammals makes them ideal alternatives to synthetic insecticide and repellent technologies. The evolutionary arms-race between plants and herbivorous insects has produced numerous plant compounds that are toxic, repellent, and modulatory to the behavior of insects [72]. Many secondary plant compounds are also considered important due to their low residual character in the environment. Their lack of persistence on treated surfaces, soil, or on human skin makes many of them ideal, safe alternatives to the currently utilized chemistries that are not readily labile in the environment. While this quality can be considered valuable for their potential risk to the environment or individuals, it may also be considered a hurdle that must be overcome in the identification and deployment of long-lasting repellent technologies. Surfaces treated with various plant compounds were in some cases no longer capable of producing appreciable repellency after 30 min [73]. This represents a need for improvement in the repellency of these various botanical compounds. 

**Figure 2:**
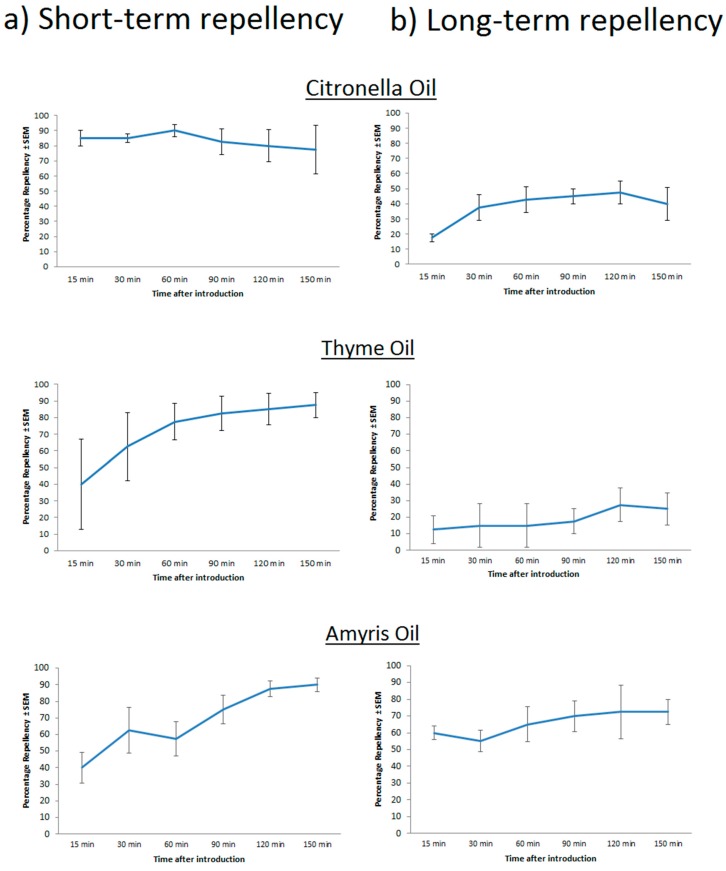
The relative spatial repellency of various plant essential oils at points immediately after treating a surface and after a five hour waiting period. Adult mosquitoes are placed in a closed 2-ft × 90-mm diameter cylinder, with one side containing a filter paper treated with the plant essential oil of interest. Relative mosquito abundance on both sides is quantified throughout the experimental interval to calculate percentage repellency. (**a**) Corresponds to the repellency caused by the plant essential oils if mosquitoes are immediately exposed to the filter paper after treatment and (**b**) corresponds to the repellency caused by various plant essential oils if mosquitoes are exposed to the treated filter paper after it is allowed to dry for 5 h. Oils that are predominantly composed of monoterpenoids (citronella and thyme oil) cause rapid, high levels of repellency in the short-term assay, with relatively lower values of repellency in the long-term assay. Oils that are composed primarily of repellent sesquiterpenoids cause lower immediate repellency, but maintain a higher level of repellency in the long-term assay (amyris oil).

Our group has extensively characterized the repellency of numerous plant compounds in the previous decades [74,75,76]. From these explorations, a wide range in efficacy has been identified among various plant compounds as spatial and contact repellents. Many of the most successful botanical repellents have been identified as terpenoids. Terpenoids are compounds produced via the isoprene biosynthesis and phenylpropanoid pathways in plants [77]. Great variation exists within this class which can be exploited for the development of novel repellents. Among this class of compounds, two types of terpenoids appear to be highly efficacious at repelling arthropods: monoterpenoids and sesquiterpenoids. Paluch et al. demonstrated that various sesquiterpenoids were highly effective at repelling yellow fever mosquitoes in a static air chamber designed for monitoring spatial repellency, and in other studies identified numerous monoterpenoids that were capable of repelling a large variety of arthropod pest species [73,74,77]. While many of these compounds are successful repellents, their residual character on treated surfaces is quite low. Figure 2 shows the repellency of various plant essential oils tested at multiple time points [78]. While many of these plant essential oils are efficacious if screened immediately after applying them to a treated surface (Figure 2a), their efficacy rapidly decreases if treated surfaces are allowed to dry for 5 h before being screened in our spatial repellency chamber (Figure 2b). Plant essential oils with high levels of spatial repellency after 5 h are predominantly composed of sesquiterpenoids (Amyris oil: Figure 2b), whereas oils containing predominantly monoterpenoids produce very high levels of initial spatial repellency followed by relatively lower repellent efficacy after a five hour period (citronella oil, thyme oil: Figure 2a,b). These differences are predominantly a function of the intrinsic repellency of these molecules (i.e., their ability to elicit a physiological response at an odorant receptor) and their volatility, which is dictated by their molecular weight, polarity, and the intermolecular forces among the molecules of the repellent compound or with the treated surface. Monoterpenoids, containing ten carbons, possess much lower molecular weights than sesquiterpenoids and are thus more volatile. This translates to much higher spatial repellency values in the short term, followed by low levels of repellency after significant quantities have volatilized off of a treated surface. Sesquiterpenoids, are larger molecules composed of 15 carbons and thus volatilize off of a treated surface more slowly, providing a longer lasting repellent character. These trends are crucial in understanding the repellent characteristics of various molecules within this chemical class and represent an exploitable paradigm from which novel repellent molecules may be crafted. 

Via a novel biorational approach, esters of monoterpenoid alcohols and acids have been created by coupling them to small molecular weight carboxylic acids and alcohols, respectively. The goal of this project has been to increase the long-lasting character of the highly repellent monoterpenoid compounds by increasing their molecular weight or polarity [78]. Numerous derivatives have been synthesized with this approach and many compounds possess higher percentage short-term and long-term repellency than the original starting monoterpenoid. Table 1 highlights the relative short-term and long-term repellency of a select monoterpenoid (citronellol) and various esters of this compound at various time points. A large number of compounds have been identified to cause significantly higher short-term and long-term repellency than citronellol itself. While modulating the molecular weight of these compounds represents a valuable approach in the development of future repellent compounds, it is only one means by which the repellency of these compounds may be improved. 

Moreover, the identification of which odorant receptors are responsible for the perception of these botanical repellents could further allow for the biorational synthesis of future repellents. Currently, technologies are being developed to rapidly screen various odorant binding receptors for their ability to recognize and respond to various repellents. The use of empty neuron systems that are capable of expressing heterologous odorant receptors in odorant neurons may be a viable method for screening numerous repellent molecules. This approach has been utilized to deorphanize a number of mosquito odorant receptors from *An. gambiae* [59]. A number of odorant receptors isolated from *An. gambiae* responded to botanical compounds and may suggest that other related compounds may also bind to these receptors. Future screening studies coupled to quantitative structure-activity relationship studies could represent a promising method by which research groups could correlate repellent potency to physicochemical characteristics of biorational molecules. These and other novel technologies aimed at identifying the odorant receptors that respond to specific terpenoids may lead to a better understanding of the relative intensity of these nervous responses. This may further elucidate which chemical properties are most conducive to the production of highly efficacious biorational repellent molecules. This approach coupled with a rational modulation of the volatility of these molecules may in turn produce some of the most repellent compounds to date.

## 7. Conclusions

Repellent technologies are important tools in the arsenal for preventing the spread of mosquito-borne disease. Current and future resistance to various repellent technologies in wild mosquito populations, lack of user compliance in adequate and timely repellent reapplication, and low residual character of current repellent technologies all represent a significant impetus for the development of novel repellent technologies. Spatial repellents represent a novel approach that could provide long-lasting repellency without the need for continual reapplication of formulations to human skin. Within this class, botanical and biorational repellents are diverse and are promising alternatives to synthetic pyrethroid spatial repellents, which are largely ineffective against pyrethroid-resistant mosquito populations. Botanical repellents are numerous and target a wide variety of odorant receptors and physiological targets, suggesting that the potential for resistance to these chemistries is sufficiently low. Current and future technologies directed toward the development of long-lasting botanical or biorational repellents could lead to promising alternatives to repellent formulations that are currently on the market. Via the optimization of repellent character by promoting binding of these compounds to odorant receptors on the mosquito antennae and lowering their volatility, a logical and exploitable paradigm exists for the development of new repellent formulations that could be deployed as spatial repellents in integrated pest management strategies throughout the world.

## Figures and Tables

**Table 1:** Relative short-term and long-term repellency of various biorational repellent esters. A repellent monoterpenoid causes relatively high percentage repellency immediately after filter papers are treated and mosquitoes are subsequently exposed. This repellency is drastically diminished if filter papers are allowed to dry for 5 h before being introduced into the testing chamber (time points 315–450 min). By derivatizing monoterpenoids via various biorational approaches, we have developed numerous compounds that are effective in both the short-term and long-term assay, potentially indicating their residual character on treated surfaces. Some compounds were even capable of causing immobilization or knockdown (KD) of exposed mosquitoes. These results may indicate that not only are these repellents highly efficacious but could represent compounds that are much more repellent than chemistries currently being utilized in current repellent technologies.

Natural and Biorational Compounds	Minutes after Treating Filter Paper					
	15	90	150	315	390	450
monoterpenoid	32.5	80	87.5	30	21.5	30
derivative 1	10	60	62.5	30	30	40
derivative 2	22.5	92.5	90	70	80	65
derivative 3	KD	KD	KD	27.5	40	55
derivative 4	25	90	92.5	27.5	17.5	20
